# Fish Galectin8-Like Exerts Positive Regulation on Immune Response Against Bacterial Infection

**DOI:** 10.3389/fimmu.2020.01140

**Published:** 2020-06-26

**Authors:** Jinzhong Niu, Yu Huang, Xinchao Liu, Fenglei Wu, Jufen Tang, Bei Wang, Yishan Lu, Jia Cai, Jichang Jian

**Affiliations:** ^1^Guangdong Provincial Key Laboratory of Pathogenic Biology and Epidemiology for Aquatic Economic Animal, Key Laboratory of Control for Disease of Aquatic Animals of Guangdong Higher Education Institutes, Southern Marine Science and Engineering Guangdong Laboratory, College of Fishery, Guangdong Ocean University, Zhanjiang, China; ^2^Laboratory for Marine Biology and Biotechnology, Qingdao National Laboratory for Marine Science and Technology, Qingdao, China; ^3^Guangdong Provincial Engineering Research Center for Aquatic Animal Health Assessment, Shenzhen, China; ^4^Guangxi Key Lab for Marine Natural Products and Combinational Biosynthesis Chemistry, Guangxi Beibu Gulf Marine Research Center, Guangxi Academy of Sciences, Nanning, China

**Keywords:** galectin-8, Nile tilapia, monocytes/macrophages, *Streptococcus agalactiae*, immune response

## Abstract

Galectin-8 is a member of the galectin family that is involved in immune response against pathogens. However, the roles of fish galectin-8 during pathogen infection require comprehensive studies. In this study, a galectin-8 homolog (OnGal8-like, OnGal8-L) was characterized from Nile tilapia (*Oreochromis niloticus*), and its roles in response to bacterial infection were analyzed. The *OnGal8-L* contains an open reading frame of 891 bp, encoding a peptide of 296 amino acids with two CRD regions of tandem-repeat galectin and two carbohydrate recognition sites. The *OnGal8-L* protein shares 46.42% identities with reported *Oreochromis niloticus* galectin-8 protein. Transcriptional expression analysis revealed that OnGal8-L was constitutively expressed in all examined tissues and was highly expressed in spleen. The transcript levels of OnGal8-L were up-regulated in the spleen, head kidney, and brain, following *Streptococcus agalactiae* (*S. agalactiae*) challenge. Further *in vitro* analysis indicated that the recombinant protein of OnGal8-L (rOnGal8L) could agglutinate erythrocyte, *S. agalactiae*, and *A. hydrophila* and bind *S. agalactiae, A. hydrophila*, and various PAMPs (lipopolysaccharides, lipoteichoic acid, poly I:C, peptidoglycan, galactose, mannose, and maltose). Also, rOnGal8L could regulate inflammatory-related gene expression, phagocytosis, and a respiratory burst of monocytes/macrophages. Moreover, *in vivo* analysis showed that OnGal8-L overexpression could protect *O. niloticus* from *S. agalactiae* infection through modulating serum antibacterial activity (AKP, ACP, and LZM), antioxidant capacity (CAT, POD, and SOD), and monocyte/macrophage proliferation and cytokine expression, as well as reducing bacterial burden and decreasing tissue damage. Our results collectively indicate that OnGal8-L plays important regulatory roles in immune response against bacterial infection.

## Introduction

Galectins are a family of proteins that possess high affinity for β-galactosides, which play a variety of roles in cell–cell adhesion, cell–matrix interaction, and transmembrane signaling (1, 2). Galectins can be found in cytoplasm, nucleus, and extracellular space (3). Presently, galectins serve as pattern recognition receptors (PRR) that regulate the innate immune processes triggered by pathogen-associated molecular patterns (4, 5). On the other hand, the intracellular galectins are involved in cellular functional processes including pre-mRNA splicing, cell growth regulation, and cell cycle processes (6, 7). Until now, 15 galectins have been discovered in mammals, which are subdivided into three subfamilies based on their conserved carbohydrate recognition domain (CRD): “prototype,” “chimera,” and “tandem repeat” (8).

Galectin-8 belongs to the tandem-repeat subfamily, consists of two CRDs linked by a peptide. Human galectin-8 has six isoforms encoded by 14 alternatively spliced transcripts (9). As a secreted protein, the immobilized galectin-8 can promote cell adhesion via attaching and aggregating integrin receptors on the cell surface, which subsequently trigger integrin-mediated signaling cascades (10). Furthermore, the involvement of galectin-8 in host defenses against bacterial infection has been well-described in mammals. The mammalian galectin-8 can detect the bacterial invasion by monitoring the integrity of endosomes and lysosomes, and then activate antibacterial autophagy to protect cells from bacterial infection (11).

To date, galectin-8 homologs have been identified in many species, but the functions of teleost galectin-8 are not well-understood yet (12). Nile tilapia (*Oreochromis niloticus*) is one of the most economically important fish all around the world (13). In recent years, the outbreak of bacterial disease has resulted in massive losses of tilapia culture (14). Although the involvement of galectin-8 in bacterial infection has been recorded in Nile tilapia (*Oreochromis niloticus*) (15), the exact roles of this molecular during bacterial infection still need to be clarified. In this study, a new tandem-repeat galectin-8 isoform (*OnGal8-L*) was identified and characterized from *O. niloticus*. The regulatory roles of OnGal8-L in immune response against bacterial infection were also investigated *in vitro* and *in vivo*. Our data will provide new insights into the function of fish galectin-8 against bacterial infection.

## Materials and Methods

### Fish Preparation and Bacterial Challenge

Nile tilapia (50 ± 10 g) were acquired from a local fish farm in Zhanjiang, Guangdong, China. The fish were cultured in a 1,000-L tank with aerated freshwater under 28 ± 2°C for 3 weeks (16). All experiments were conducted according to the principles and procedures of Guangdong Province laboratory animal management regulations (17).

To explore the tissue distribution of OnGal8-L in healthy tilapia, the tissues including brain, head kidney, gill, spleen, thymus, heart, liver, muscle, skin, and intestine were collected and frozen quickly by liquid nitrogen and stored at −80°C until use.

*S. agalactiae* (ZQ1901) used in the experiment was isolated from Nile tilapia and kept in our laboratory (18). The *S. agalactiae* was dissolved in phosphate-buffered saline (PBS) with a final concentration of 1 × 10^7^ cells/mL. The stimulation groups were injected with 100 μL *S. agalactiae*. The samples including spleen, head kidney, and brain were collected at seven time points (0, 4, 12, 24, 48, 72, and 96 h), and then stored at −80°C until use.

### Stimulation of *S. agalactiae* on Head Kidney-Derived Monocytes/Macrophages

Monocytes/macrophages were prepared as previous studies (18, 19). Briefly, healthy tilapia was anesthetized by MS222. The head kidney was carefully excised and transferred through a 40-μm stainless nylon mesh (Greiner Bio-OneGmbH, Germany). The cell suspension was suspended in Dulbecco's Modified Eagle Medium (DMEM) (Gibco, US) supplemented with 100 U/mL penicillin, 100 μg/mL streptomycin, and 25 U/mL heparin (Gibco). The cell suspension was slowly added into a 51%/34% percoll (GE Healthcare) density gradient, centrifuged at 400 *g* for 40 min, and the cell layer of the interface was carefully aspirated. The harvested cells were washed with PBS and collected again through centrifugation at 500 g for 10 min. Cell viability was measured using a Trypan Blue Staining kit (Sangon Biotech). The cells were cultured at 25°C for 24 h and then removed the non-adherent cells.

Challenge experiments were carried out according to the method of Mu et al. (20). The monocytes/macrophages were stimulated by formalin-inactivated *S. agalactiae* (1 × 10^7^ cells/mL). The cells were collected and lysed by Trizol Reagent (Sangon Biotech) at 0, 3, 6, 12, 24, and 48 h post-challenge, respectively.

### Cloning and Sequence Analysis of *OnGal8-L*

In order to obtain the open reading frame of OnGal8-L, total RNA from spleen was extracted by using EasyPure RNA Kit (TransGen, China) according to the manual protocol. The first-stand cDNA was synthesized from total RNA by using EasyScript One-Step gDNA Removal and cDNA Synthesis SuperMix (TransGen, China). The specific primers (OnGal8-L-F, OnGal8-L-R) were designed based on Nile tilapia transcriptome data (https://www.ncbi.nlm.nih.gov/bioproject/PRJNA244908) and the prediction of NCBI Basic Local Alignment Search Tool. All the primers used in this study were designed by Primer 6.0 and showed in Table 1.

**Table 1:** Primers used in this study.

**Primers**	**Nucleotide sequence(5^′^ → 3^′^)**	**Comment**
OnGal8-L-F	ATGTCCGTGGCTAAC	ORF amplification
OnGal8-L-R	TCACCACAGCTTGATGTC	ORF amplification
M13F	TGTAAAACGACGGCCAGT	Sequencing
M13R	CAGGAAACAGCT ATGACC	Sequencing
qOnGal8-L-F	CTCCACCTGAACCCCCGC	RT-PCR
qOnGal8-L-R	CCCGCTCCTCCTGACCCC	RT-PCR
β-actin-F	CGAGAGGGAAATCGTGCGTGACA	RT-PCR Control
β-actin-R	AGGAAGGAAGGCTGGAAGAGGGC	RT-PCR Control
EOnGal8-L-F	CGCGGATCCTCCGTGGCTAAC	Protein expression
EOnGal8-L-R	CCGCTCGAGCCACAGCTTGATGTC	Protein expression
qIL-6-F	ACAGAGGAGGCGGAGATG	RT-PCR
qIL-6-R	GCAGTGCTTCGGGATAGAG	RT-PCR
qIL-8-F	GATAAGCAACAGAATCATTGTCAGC	RT-PCR
qIL-8-R	CCTCGCAGTGGGAGTTGG	RT-PCR
qIL-10-F	TGGAGGGCTTCCCCGTCAG	RT-PCR
qIL-10-R	CTGTCGGCAGAACCGTGTCC	RT-PCR
qMIF-F	CACATCAACCCTGACCAAAT	RT-PCR
qMIF-R	GCCTGTTGGCAGCACC	RT-PCR
pEGFP-Gal8-L-F	CCGCTCGAGCT TCCGTGGCTAAC	Overexpression
pEGFP-Gal8-L-F	CGGGATCCCCACAGCTTGATGTC	Overexpression
qIL-1β-F	AAGATGAATTGTGGAGCTGTGTT	RT-PCR
qIL-1β-R	AAAGCATCGACAGTATGTGAAAT	RT-PCR
qTNF-α-F	AGGGTGATCTGCGGGAATACT	RT-PCR
qTNF-α-R	GCCCAGGTAAATGGCGTTGT	RT-PCR

*The restriction sites used in this study are underlined*.

The potential open reading frame of OnGal8-L was analyzed with ORF finder program (https://www.ncbi.nlm.nih.gov/orffinder/). Molecular weight, theoretical pI, amino acid composition of OnGal8-L were predicted by ProtParam tool (https://web.expasy.org/protparam/). Multiple sequence alignment of OnGal8-L protein sequences was performed by Clustal W program (http://www.clustal.org/clustal2/). The similarity analyses of the determined amino acid sequence were performed by UniProt programs (https://www.uniprot.org/). Phylogenic trees were constructed by the neighbor-joining method using MEGA X software with 1,000 bootstrap replications. The evolutionary distances were computed using the Poisson correction method (21) and are in the units of the number of amino acid substitutions per site.

### Quantitative Real-Time PCR of *OnGal8-L*

The RNA from 10 different tissues (heart, brain, spleen, liver, thymus, head kidney, intestine, muscle, gill, and skin) of healthy tilapia were extracted and then reverse-transcribed into cDNA, as described in section Stimulation of *S. agalactiae* on Head Kidney-Derived Monocytes/Macrophages. The β-actin (housekeeping gene) was chosen as the internal reference gene. Quantitative RT-PCR (qRT-PCR) was carried out on Roche LC384 Lightcycler^TM^ (Roche, Switzerland) with a PCR reaction volume of 10 μL containing 5 μL of FastStart Essential DNA Green Master (Roche, Switzerland), 0.5 μL of each primer (2 μM), and 3.5 μL of nuclease-free water. The program was performed as follows: 1 cycle of 10 min at 95°C, 40 cycles of 10 s at 95°C, 15 s at 55°C, and 15 s at 72°C. The relative expression level of *OnGal8-L* mRNA were calculated using 2^−ΔΔCt^ method (22).

### Preparation of OnGal8-L Recombinant Protein (rOnGal8L)

The specific primers (EOnGal8-L-F and EOnGal8-L-R) with restriction sites (*BamH* I and *Xho* I) were designed (Table 1) to amplify OnGal8-L ORF. The PCR product was purified and ligated into the pMD18-T vector. The recombinant plasmids pMD-18T-OnGal8-L and pGEX-4T-1 were digested with *BamH* I and *Xho* I. The digested products were ligated and transformed into *E. coli* BL21 (DE3) (TransGen, China). The positive clone was verified by PCR and DNA sequencing. Then, the positive clone was picked to culture in fresh LB liquid medium containing ampicillin (100 μg/mL). When OD_600_ reached 0.4–0.6, isopropyl-β-Dthiogalactopyranpside (IPTG) was added into cultured bacteria with a final concentration of 1 mmol/L. The IPTG added cultures were continuously induced at 37°C for 5 h. The bacteria were collected and washed three times with PBS. Lysozyme was added into bacterial solution with a final concentration of 1 mg/mL, and placed on ice for 30 min, then centrifuged at 4°C for 10 min. The supernatant was purified using a GST-tag protein purification kit (Beyotime, China), desalted and concentrated using an Amicon Ultra Centrifugal Filter (Amicon, USA). The purified protein was analyzed by 10% reducing SDS-PAGE and Western blot. The empty vector (pGEX-4T-1)-expressed GST-tagged protein was prepared as described above and used as control in further analysis.

### Hemagglutination Assay

The hemagglutination assay was performed according to the method of Madusanka et al. (12). Briefly, the blood of Nile tilapia was collected in a centrifuge tube containing 1% sodium heparin. After centrifuging at 2,000 g for 15 min, the supernatant was removed and the collected erythrocytes were washed four times with sterile TBS. The harvested erythrocytes were adjusted to 1 × 10^5^ cells/mL with sterile TBS. Then, a 500-μL erythrocyte suspension was incubated with 10 μL of rOnGal8L (1 mg/mL) at room temperature for 10 min. Both PBS and the GST-tag protein were used as control. The incubated erythrocytes were transferred onto slide, and the agglutination was observed by fluorescence microscope.

### Bacterial Agglutination Assay

Briefly, *S. agalactiae* and *A. hydrophila* were cultured until the OD_600_ reached 0.4–0.6; the bacteria were collected and washed three times with PBS. The collected bacteria were resuspended in 0.1 M Na_2_CO_3_. Then FITC (Solaribo, China) was added into resuspended bacterial solution with a final concentration of 0.1 mmol/L. The FITC-added solutions were incubated at 37°C for another 30 min. After incubation, the bacteria were centrifuged three times to completely remove the FITC and incubated with 10 μL of rOnGal8L (1 mg/mL) at room temperature for 1 h. The incubated bacteria were transferred onto slide and observed with a fluorescence microscope.

### Binding Assay of rOnGal8L to Bacterial Pathogens and Carbohydrates

The binding abilities of rOnGal8L to Gram-positive bacteria (*S. agalactiae*) and Gram-negative bacteria (*A. hydrophila*) were detected by the method of Bai et al. (23). Briefly, the bacteria were cultured until the OD_600_ reached 0.4–0.6. Ten microliters of rOnGal8L (1 mg/mL) was added into 500 μL of bacterial solution and incubated at 37°C for 1 h. The incubated bacterial were collected and washed three times with PBS, then lysed the bacteria with 7% SDS for 1 min and centrifuged at 12,000 rpm for 10 min. The supernatant was collected for Western blot analysis. The anti-GST mouse monoclonal antibody (1:1,000; 1 mg/mL) (Sangon Biotech, Shanghai) was used as the primary antibody, and the HRP-goat α-mouse antibody (1: 5,000; 1 mg/mL) (Sangon Biotech, Shanghai) was used as the secondary antibody. The bacteria incubated with PBS or pGEX-4T-1 were used as control. Previous study shows that galectins bind to bacteria through the carbohydrates on bacterial cell wall, which can be competitively inhibited by another carbohydrates (24). So several saccharides including lipopolysaccharide (LPS), lipoteichoic acid (25), galactose, mannose, and maltose were chosen to explore the inhibitory capacities on the binding activity of rOnGal8L to bacterial through competitive binding assay. The rOnGal8L or GST-tag protein (1 mg/mL, 10 μL) was incubated with each saccharide (1 mg/mL, 50 μL) and 500 μL of *S. agalactiae* and *A. hydrophila* at room temperature for 1 h and incubated mixtures were examined by western blot as described above.

To further understand the binding activity of rOnGal8L to carbohydrates, enzyme-linked immunosorbent assay (ELISA) was performed according to the method of Zhang et al. (26). Briefly, LTA, LPS, Poly I:C, PGN, galactose, mannose, and maltose were chosen and adjust to a concentration of 100 μg/mL with 20× Coating Buffer (Sangon Biotech, Shanghai). The 10-fold serial dilutions of carbohydrates were made. Then the diluted carbohydrates solutions (100 μL/well) were added into 96-well-plates for coating at 4°C overnight and blocked at 37°C for 2 h in the next day. The empty wells were used as blank control. Plates were washed with TBST three times between each step, and rOnGal8L (100 μg/mL, 100 μL) was added to the wells and incubated at 37°C for 2 h after final wash. Following incubation, the anti-GST mouse monoclonal antibody (1: 1,000, 1 mg/mL) (Sangon Biotech, Shanghai) was used as the primary antibody, and the HRP-goat α-mouse antibody (1: 5,000, 1 mg/mL) (Sangon Biotech, Shanghai) was used as the secondary antibody. One-hundred milliliters of TMB mixed reaction solution was added to each well and incubated at 37°C for 15 min in the dark, then used H_2_SO_4_ (2 M, 50 μL) to terminate the reaction. The optical density (O.D.) at 405 nm was measured using a Microplate Reader (Thermo, USA).

### Bacterial Growth Inhibition Assay

The bacterial growth inhibition assay of rOnGal8L was performed as described in a previous study (27). In brief, *S. agalactiae* and *A. hydrophila* were cultured overnight, the bacterial pellet was collected by centrifugation, and then adjusted to an initial concentration of 1 × 10^5^ cells/mL with culture medium. One-hundred microliters of diluted bacterial solution was added into a 96-well-microtiter plate, the rOnGal8L, GST-tag protein, or PBS was added with a final concentration of 50 μg/mL at the same time. The plates were incubated at 37°C and the O.D. value of 600 nm were measured every 2 h. The antibacterial activities were evaluated by the bacterial growth density (O.D. value).

### Analysis of Inflammatory-Related Gene Expression in Monocytes/Macrophages After rOnGal8L Stimulation

The experiment was performed according to the method of Crinier et al. (28). Briefly, the monocytes/macrophages were stimulated with 50 μL of rOnGal8L (1 mg/mL), GST-tag protein (1 mg/mL), and PBS, respectively. The cells were collected, and the cDNA were synthesized, as described in section Cloning and Sequence Analysis of *OnGal8-L*. The expression levels of *IL-6, IL-10, IL-8*, and *MIF* (Table 1) in monocytes/macrophages were measured by qRT-PCR. Similar with the method described in section Quantitative Real-Time PCR of *OnGal8-L*, the qRT-PCR was performed on Roche LC384 Lightcycler^TM^ with a reaction volume of 10 μL. The β-actin was applied as the internal reference gene. The relative expression levels of inflammatory-related genes were calculated using 2^−ΔΔCt^ method.

### Phagocytosis Assay

Phagocytosis assay was performed as the method of Bai et al. (29). Briefly, 200 μL of FITC-labeled *S. agalactiae* and *A. hydrophila* suspension was mixed with 180 μL of monocytes/macrophages and 20 μL of rOnGal8L (1 mg/mL) and incubated in the dark for 1 h with shaking every 5 min. In the control group, 20 μL of PBS or GST-tag protein (1 mg/mL) was used instead of rOnGal8L. Then centrifuge at 500 g for 10 min to completely remove the non-ingested bacteria. The results were analyzed using flow cytometer. The fluorescence data for this experiment is limited to a gate to ensure the accuracy of the analysis. Each sample measurement was repeated in triplicate.

### Respiratory Burst Assay

The effect of rOnGal8L on the respiratory burst activity of monocytes/macrophages was performed by the methods described in the References (30–32). In brief, PMA was used to stimulate monocytes/macrophages to produce reactive oxides that induced the oxidation of dihydrorhodamine to rhodamine 123, which was capable of emitting fluorescence. According to the protocol of PMN Oxidative Burst Quantitative Assay Kit (Absin Bioscience, Shanghai), 300 μL of monocytes/macrophages cells (2 × 10^7^ cells/mL) were mixed with 10 μL of rOnGal8L (1 mg/mL) and incubated at 25°C for 1 h. Then 50 μl of PMA was added and the mixture was incubated at 37°C for 15 min. Subsequently, 25 μL of dihydrorhodamine was added to the mixture and incubated at 37°C for 5 min in the dark. One milliliter of diluted hemolysin was added at room temperature and hemolyzed for 15 min. The mixture was washed twice with PBS, centrifuged at 1,500 rpm for 5 min and the supernatant was discarded. Then the cells were resuspended in 0.5 mL PBS for flow cytometry analysis. The experimental procedures of control groups (GST-tag protein or PBS) were the same as described above.

### Plasmid Construction and Overexpression *in vivo*

Per the method described in section Preparation of OnGal8-L Recombinant Protein (rOnGal8L), pEGFP-C1 eukaryotic expression vector was constructed using specific primers (Table 1). The recombinant plasmid pEGFP-Gal8-L and pEGFP-C1 were extracted using E.Z.N.A. Endo-free Plasmid Mini Kit (Promega, USA) in compliance with manufacturer's instructions and then used for further analysis.

According to the *in vivo* overexpression protocol described in Huang et al. (33), the plasmids pEGFP-Gal8-L and pEGFP-C1 were adjusted to a final concentration of 200 μg/mL and 50 μl of each diluted plasmid was injected intramuscularly into fish. PBS group was set as a control. After 7 days post-injection, the spleen of each group was collected and fixed using standard histological techniques. The fluorescence signal in collected spleens were detected by fluorescence microscope (LEICA, CTR6000, Germany).

### Analysis of the Regulatory Roles of OnGal8-L Overexpression on Immune Response *in vivo*

Fish blood samples from each group were collected from caudal vein with a sterile syringe at day 7 after plasmid (pEGFP-Gal8-L and pEGFP-C1) and PBS administration. The serum was isolated for the detection of non-specific immune parameters, including alkaline phosphatase activity (AKP), acid phosphatase (ACP), lysozyme (LZM), catalase (CAT), peroxidase (POD), and superoxide dismutase (SOD) using the corresponding protease detection kit (Nanjing Jiancheng, Bioengineering Institute, China). At day 7 after plasmids and PBS administration, the fish were infected intramuscularly with 100 μl of *S. agalactiae* resuspended in PBS (1 × 10^7^ cells/mL). After bacterial stimulation, three tissues samples including head kidney, spleen, and liver were obtained from each group fish at 3, 6, 12, 24, 48, 72, and 96 hour post injection (hpi), respectively. The qRT-PCR was conducted *per section* Cloning and Sequence Analysis of *OnGal8-L* to test the express level of immune-related genes, including *IL-10, IL-1*β, and *TNF-*α (Table 1).

### Determination of the Effects of OnGal8-L Overexpression on Bacterial Resistance *in vivo*

As the challenge experiment design in section Analysis of the Regulatory Roles of OnGal8-L Overexpression on Immune Response *in vivo*, the bacterial burden in tilapia tissues of different treatment groups were evaluated after bacterial infection. The bacterial counts were carried out as the method described in the References (34). Briefly, the spleen, head kidney, and liver were collected from the experimental fish at 24 hpi. After weighing, the collected tissues were homogenized in 1 mL of sterile PBS. The homogenized tissues were serially diluted and spread on brain–heart infusion agar plates and cultured at 37°C for 24 h. Single colonies were identified by PCR using *S. agalactiae*-specific primers. Finally, colony-forming units (CFU) in all plates were counted and multiplied by the dilution factor. For histological observation, various tissues including the head kidney, spleen, and liver were collected at 24 h after bacterial injection. The histological changes of these samples were observed under microscope.

The survival assay was performed according to the method described in our previous study (33). Briefly, 160 fish were divided into four groups and injected with plasmids (pEGFP-Gal8-L or pEGFP-C1) and PBS. At day 7 post injection (p.i.), fish in each group were challenged with activated *S. agalactiae* at 1 × 10^8^ colony-forming units (CFU)/fish by intraperitoneal injection. Daily statistical morbidity lasted for 10 days, and the survival rate (SR) was calculated as SR = (surviving fish÷40) × 100%.

### Statistical Analysis

All data in this study were displayed as means ±standard deviation (SDs). Statistical analysis was performed by the least significant difference (LSD) test using SPSS 17.0 software. Difference between two groups was considered as significant at ^*^p < 0.05 or highly significant at ^**^p < 0.01.

## Results

### Sequence Analysis of *OnGal8-L*

**Figure 1:**
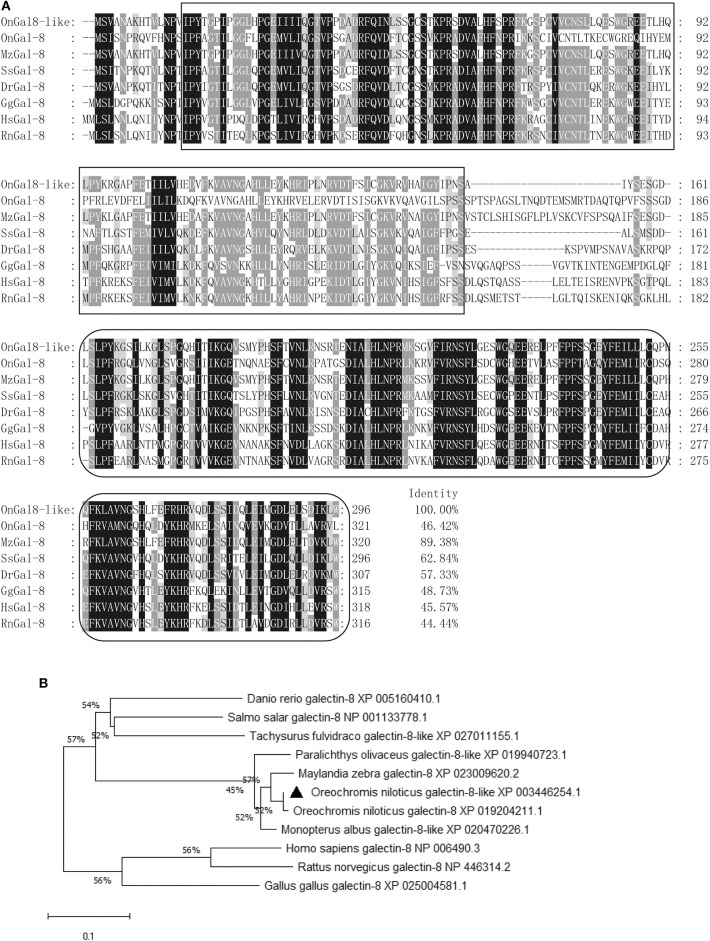
**(A)** Multiple sequence alignment of galectin-8(like) from various species. Conserved amino acid residues are shaded dark gray, and similar amino acids are shaded light gray. The CRDs are in two black boxes. Conserved residues (H-N-R, V-N, and W-E-R) for carbohydrate recognition and binding are shown above the alignment sequence. The GenBank accession numbers of genes involved are as below: *M. zebra* galectin-8, XP_023009620.2; *S. salar* galectin-8, NP_001133778.1; *D. rerio* galectin-8, XP_005160410.1; *G. gallus* galectin-8, NP_001010843.1; *O. niloticus* galectin-8, XP_019204211.1; *H. sapiens* galectin-8, NP_006490.3; and *R. norvegicus* galectin-8, NP_446314.2; **(B)** Phylogenetic tree of OnGal8-like family members constructed using the NJ method by MEGA X program based on the alignment of 10 members of the Gal-8 group. The numbers at each branch indicates the bootstrap values (%).

The ORF of OnGal8-L was 891 bp in length and encoded 296 amino acids. The predicted molecular mass of OnGal8-L was 33.14 KDa and the theoretical pI was 5.03. It had two CRD domains (N- and C-CRD domains) and a carbohydrate recognition site. Multiple sequence alignment of OnGal8-L and other reported galectin-8 found that OnGal8-L possessed conservative sequences of the galectin family such as V-N, H-N-RL, and W-E-R in two CRDs, which shared higher identities with known galectin-8. While the linker peptide between two CRDs in OnGal8-L was much shorter than that in galectin-8 (Figure 1A). Phylogenetic analysis showed that OnGal8-L located within fish galectin-8 subgroup and was clustered close to *Oreochromis niloticus* galectin-8 and *Maylandia zebra* galectin-8 (Figure 1B).

### The Expression Profiles of OnGal8-L *in vivo* and *in vitro*

**Figure 2:**
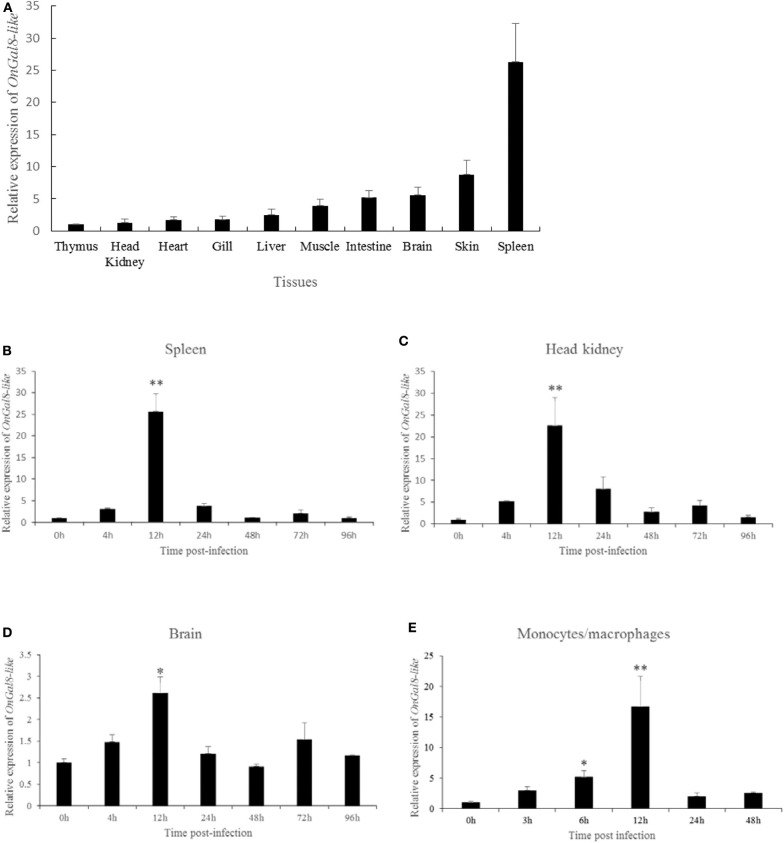
**(A)** Tissue distribution of OnGal8-L mRNA in healthy Nile tilapia. The ratio refers to the gene expression in different tissues relative to that in thymus, and target gene expression is normalized to β*-actin*. The results were mean ± SD of three replicate measurements. **(B–E)** Expression analysis of OnGal8-like in spleen **(B)**, head kidney **(C)**, brain **(D)**, and monocytes/macrophages **(E)** after *S. agalactiae* challenge was performed by qRT-PCR. Data are shown as mean ± SD from three replicate measurements. Significant difference was indicated by asterisks as *0.01 < *P* < 0.05 and **P < 0.01.

The tissue distribution of OnGal8-L was investigated by qRT-PCR analysis. The results showed that the relatively higher transcriptional levels of *OnGal8-L* were observed in the spleen, skin, brain, and intestine; followed by muscle, liver, gill, heart, and head kidney; and the lowest level in thymus (Figure 2A).

After *S. agalactiae* infection, *OnGal8-L* transcripts increased in a time-dependent pattern and reached its peak at 12 h (Figures 2B–D) in head kidney, spleen, and brain. Additionally, the transcriptional levels in head kidney and spleen were much higher than that in brain. Similarly, the expression of *OnGal8-L* in monocytes/macrophages was also induced by bacterial stimulation and reached the peak at 12 h (16.35-fold) post-stimulation (Figure 2E).

### Purification and Western Blotting Analysis of rOnGal8-L

**Figure 3:**
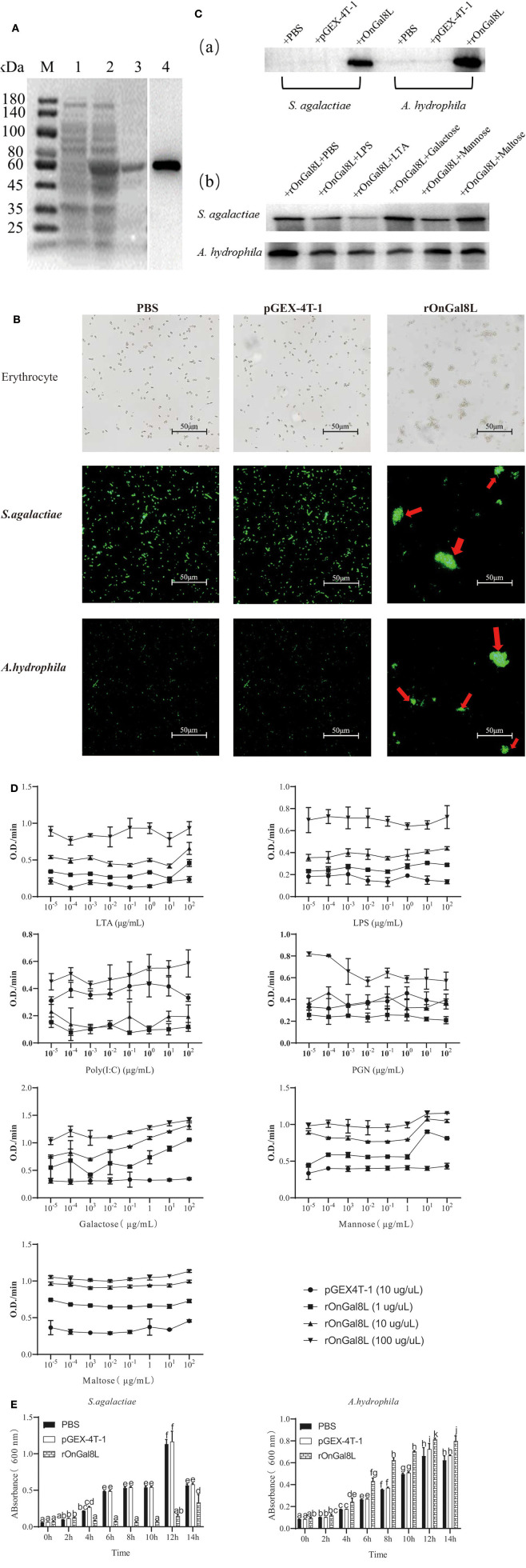
**(A)** SDS-PAGE and Western blot of rOnGal8L. Lane M: markers (25–180 kDa); lane 1: bacteria liquid before IPTG induction; lane 2: bacteria liquid after IPTG induction; lane 3: purified rOnGal8L; lane 4: Western blot analysis of rOnGal8L. **(B)** Agglutinating activity of rOnGal8L against fish erythrocytes, FITC-labeled *S. agalactiae* and *A. hydrophila*. PBS or pGEX-4T-1 was incubated with bacteria as a negative control. The agglutination was presented with red arrows; **(C)** a: Western blot analysis of rOnGal8L binding to *S. agalactiae* and *A. hydrophila*; b: Western blot analysis of the inhibitory effect of carbohydrate on the binding of rOnGal8L to bacteria. **(D)** ELISA analysis of rOnGal8L incubated with LTA, LPS, poly I:C PGN, galactose, mannose, and maltose in different concentration, respectively. The 10-fold serial dilutions of carbohydrates were made and the diluted carbohydrates solutions (100 μL/well) were added into 96-well-plate. The empty wells were applied as blank control. **(E)** The bacteriostatic activity of rOnGal8L. The growth curve of *S. agalactiae* and *A. hydrophila*. The bacteria were incubated with rOnGal8L, pGEX-4T-1, and PBS, respectively. OD_600_ was measured at different time points. Data are shown as mean ± SD. The error bars represent standard deviation (*n* = 3), and different letters (a–c) depict statistical significance between different groups (*p* < 0.05).

The prokaryotic recombinant vector pGEX-4T-1-OnGal8-L was transformed into *E. coli* and induced to produce the OnGal8-L recombinant protein (rOnGal8-L). As predicted, an OnGal8-L recombinant protein of 59 KDa was obtained (Figure 3A, lane 2 and 3). Western blot showed that the protein could be specifically recognized by GST-tagged proteins (Figure 3A, lane 4), indicating that the recombinant protein was successfully expressed.

### Agglutinating Activity of rOnGal8L to Erythrocytes and Bacterial Pathogens

To clarify the agglutination ability, rOnGal8L was used to incubate with erythrocytes, *S. agalactiae* and *A. hydrophila*. The strong agglutination activities of rOnGal8L on these three cells were seen, while no agglutinations were observed in the pGEX-4T-1 group and the PBS group (Figure 3B).

### Binding Assay of rOnGal8L to Bacterial Pathogens and Carbohydrates

For the determination of the binding ability of rOnGal8L to bacteria, the *S. agalactiae* and *A. hydrophila* were incubated with rOnGal8L and then lysed with 7% SDS as described above (23). The supernatant after centrifugation was collected for Western blotting analysis. The results showed that rOnGal8L could strongly bind to both chosen bacteria (Figure 3Ca). Then, different polysaccharides were added to perform competitive binding experiments on bacteria. The results indicated that LTA reduce the binding of rOnGal8L to bacteria, while no obvious inhibitory effects were found on the other sugars (Figure 3Cb).

The results described above showed that different polysaccharides could affect the binding capacity of rOnGal8L, so we used ELISA to detect the binding capacities of rOnGal8L to carbohydrates. As shown in Figure 3D, only the highest concentration rOnGal8L (100 μg/μL) exhibited stable binding activity to carbohydrates of each concentration, which showed strongest binding activity to galactose, followed by maltose, mannose, LTA, LPS, PGN, and poly I:C. In addition, the binding activities of highest concentration of rOnGal8L to polysaccharides increased with the increasing carbohydrates concentration except that to PGN.

### Inhibitory Effect of rOnGal8L on Bacterial Growth

After incubating with rOnGal8L, the growth of the *S. agalactiae* and *A. hydrophila* were monitored for 14 h. The results showed that rOnGal8L significantly suppressed the growth of *S. agalactiae*, but not *A. hydrophila* (Figure 3E).

### Effects of rOnGal8L on the Activity of Monocytes/Macrophages

**Figure 4:**
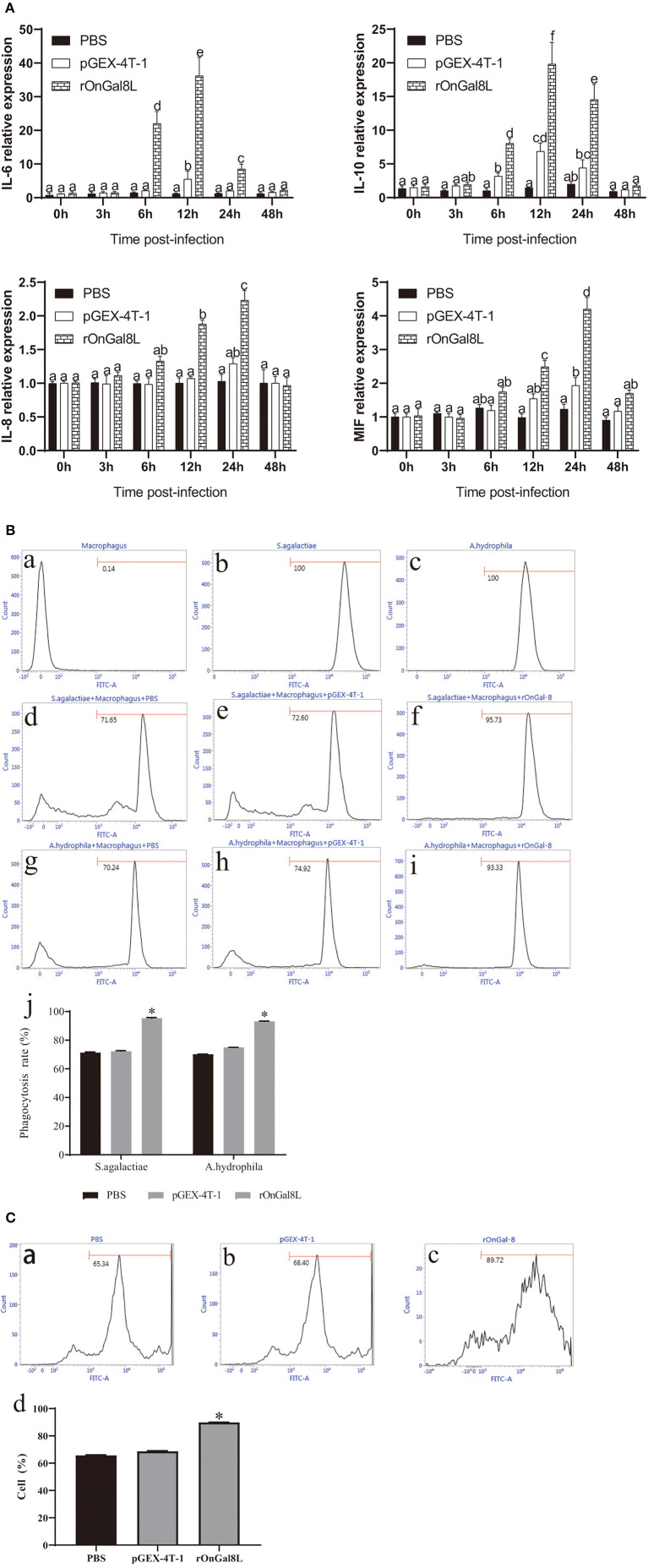
**(A)** The mRNA expressions of *IL-6, IL-10, IL-8*, and *MIF* in the head kidney-derived monocytes/macrophages. The monocytes/macrophages were treated with PBS, pGEX-4T-1 (50 μg/mL), and rOnGal8L (50 μg/mL). The mRNA levels of *IL-6, IL-8, IL-10*, and *MIF* genes were normalized to that of β*-actin* gene. **(B)** a–c: The effects of rOnGal8L on phagocytosis activity of head kidney-derived macrophages. d–i: The histogram of macrophages, FITC-labeled *S. agalactiae*, and *A. hydrophila*. The histogram of flow cytometric analyses of the macrophages phagocytosing *S. agalactiae* and *A. hydrophila* after pre-incubated with PBS, pGEX-4T-1, and rOnGal8L, respectively. j: The histogram of the phagocytosis rates. The average standard deviation was obtained from three replicate tests. Values were shown as mean ± SD (*n* = 3; *p < 0.05). **(C)** a–c: Flow cytometric analysis of the respiratory burst of head kidney-derived monocytes/macrophages. The histogram of the respiratory burst activities of monocytes/macrophages after pre-incubated with PBS (a), pGEX-4T-1 (b), or rOnGal8L (c) (10 μg/mL). (d): The histogram of the positive cell rates. The average standard deviation was obtained from three replicate tests. Values were shown as mean ± SD (*n* = 3; *p < 0.05).

To determine the modulatory effects of rOnGal8L on the activity of monocytes/macrophages, several inflammatory-related genes, including *IL-6, IL-10, IL-8*, and *MIF*, were chosen for qRT-PCR analysis. After rOnGal8L incubation, the chosen genes in rOnGal8L-treated group were significantly up-regulated. Compared to PBS-treated group, the *IL-6* and *IL-10* of rOnGal8L-treated group reached their peak at 12 h and showed higher expression levels (*IL-6*, 36.2- vs. 5.5-fold, 12 h; *IL-10*, 19.8- vs. 6.8-fold, 12 h), while *IL-8* and *MIF* reached their peak at 24 h. Moreover, rOnGal8L could significantly enhance the phagocytosis of macrophages to *S. agalactiae* and *A. hydrophila* (Figure 4B). The enhancement of rOnGal8L on respiratory burst of monocytes/macrophages was also seen (Figure 4C).

### OnGal8-L Overexpression Improved Serum Non-specific Immunological Parameters

**Figure 5:**
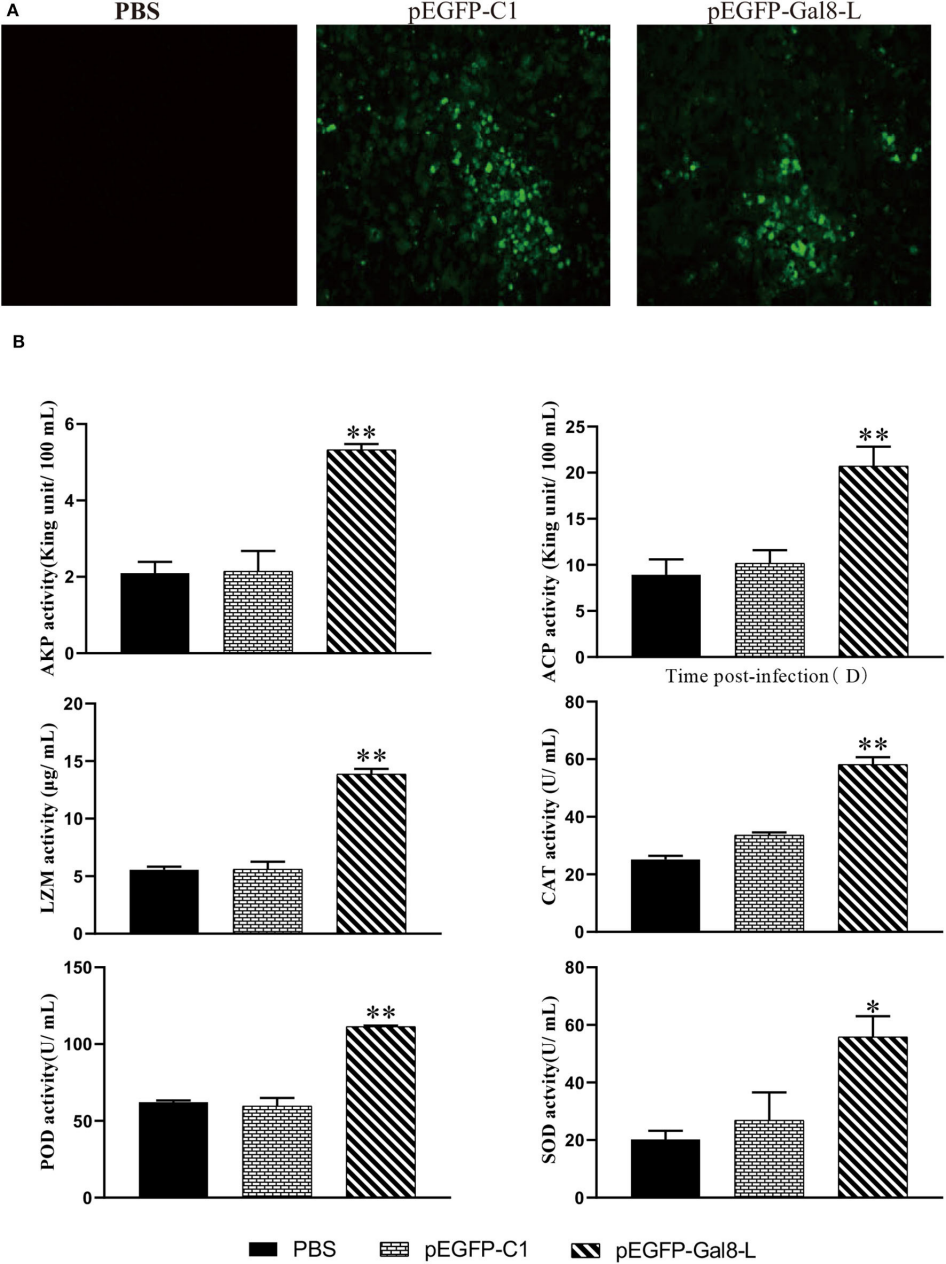
**(A)** Determination of fluorescence signals in pEGFP-Gal8-L- or pEGFP-C1-administered tilapia. Fish were injected with PBS, pEGFP-C1, and pEGFP-Gal8-L. Spleen tissue was obtained at day 7 after plasmid administration and examined with a fluorescence microscope. **(B)** Changes in antioxidant activity and non-specific immune response of administered tilapia. The average standard deviation was obtained from three replicate samples (*p < 0.05; **p < 0.01).

To confirm the overexpression of OnGal8-L *in vivo*, the spleen sections were observed at 7 days after plasmid injection. The results showed that green fluorescence appeared in both pEGFP-Gal8-L group and pEGFP-C1 group, while no fluorescence was observed in the PBS group (Figure 5A). Furthermore, a series of non-specific immunological parameters were examined. The results showed that the activities of parameters including AKP, ACP, LZM, CAT, POD, and SOD in pEGFP-Gal8-L group were significantly higher than pEGFP-C1 and PBS group (Figure 5B).

### OnGal8-L Overexpression Reduced the Bacterial Burden in *S. agalactiae*-Infected Fish

**Figure 6:**
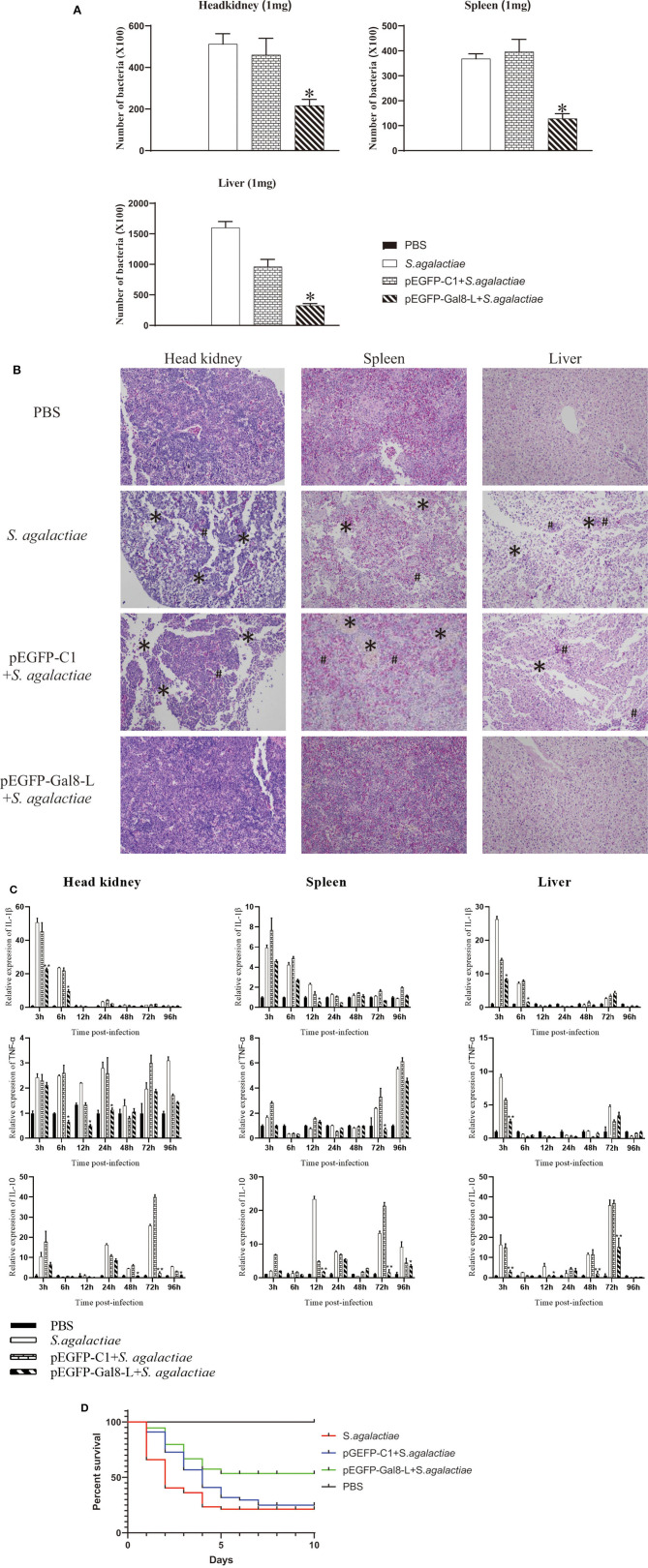
**(A)** The bacterial burden in administered tilapia after challenge with live *S. agalactiae*. Nile tilapia were administered with PBS, pEGFP-C1, or pEGFP-Gal8-L and then infected with *S. agalactiae*. Bacterial numbers in the spleen were determined at 24 hpi. Values are shown as means ± SDs (*n* = 3). **p < 0.01. **(B)** Histological observation of the head-kidney (left lane), spleen (middle lane), and liver (right lane) in administered fish after *S. agalactiae* infection. *markers, necrotic foci; #markers, severe hemorrhage. *N* = 5 each group. All sections were stained with HE and photographed under an optical microscope (×400 magnification). **(C)** qRT-PCR analysis of cytokine transcripts in head kidney, spleen, and liver of administered tilapia following *S. agalactiae* infection. Five fish/group were sampled at 3, 6, 12, 24, 48, 72, and 96 hpi, respectively. Cytokine expressions were normalized to β-actin. Data are shown as mean ± SD. Significant difference of pEGFP-Gal8-L-injected group to PBS-injected group was indicated by asterisks as *0.01 < *p* < 0.05 and **p < 0.01. **(D)** Survival rates of administered tilapia after challenge with live *S. agalactiae*. The fish were injected with PBS, pEGFP-C1, or pEGFP-Gal8-L. Fish were challenged with *S. agalactiae* (10^8^ CFU/fish), daily statistical morbidity lasted for 10 days, and the survival rate (SR) was calculated as: SR = (surviving fish÷40) × 100%; *n*= 40 each group.

The plate-counting method was applied to calculate the bacterial burden in the spleen, head kidney, and liver. The amounts of bacteria per milligram of tissues were represented by CFU. The PBS-injected group was set as the negative control, and no *S. agalactiae* was seen in the spleen and head kidney. After being challenged with *S. agalactiae*, the number of bacteria in head kidney of pEGFP-Gal8-L-injected group (2.16 × 10^4^ CFU per mg) was much lower than that in the PBS-injected group (5.12 × 10^4^ CFU/mg) and the pEGFP-C1-injected group (4.6 × 10^4^ CFU/mg). Similarly, onGal8-L overexpression significantly reduced the bacterial burden in spleen (1.28 × 10^4^ CFU/mg) compared with the PBS-injected group (3.68 × 10^4^ CFU/mg) and the pEGFP-C1-injected group (3.96 × 10^4^ CFU/mg). In liver, the amounts of bacteria in PBS-injected group was 1.6 × 10^5^ CFU/mg, the pEGFP-C1-treated group was 9.6 × 10^4^ CFU/mg, while the pEGFP-Gal8-L-injected group was significantly lower (3.24 × 10^4^ CFU/mg; Figure 6A).

### OnGal8-L Overexpression Impaired Tissue Damage Caused by *S. agalactiae* Infection

After *S. agalactiae* infection, series of marked pathological changes were seen in *S. agalactiae*-injected group and pEGFP-C1-injected group. For examples, in head kidney, there were a large amount of tissue bleeds and shed necrosis to form necrotic foci; in spleen, diffuse hyperemia and cell necrosis was found, and the integrity of the plasma membrane was disrupted; in liver, the cells were sparsely arranged, and many necrotic foci were present in the liver parenchyma, and inflammatory hyperemia appeared locally. While the similar phenomenon was not exhibited in PBS-injected group and pEGFP-Gal8-L-injected group (Figure 6B).

### Regulatory Roles of OnGal8-L Overexpression on Inflammatory-Related Gene Expression During *S. agalactiae* Infection *in vivo*

qRT-PCR was carried out to detect the expression of inflammatory-related genes in head kidney, spleen, and liver from fish in different treated groups following *S. agalactiae* infection (Figure 6C). Compared to the PBS-injected group (without *S. agalactiae* infection), the bacterial infection markedly induced the transcriptional levels of *IL-1*β (head kidney: 50.7-fold at 3 hpi; spleen: 5.9-fold at 3 hpi; liver: 26.4-fold at 3 hpi), *TNF*α (head kidney: 2.4-fold at 3 hpi; spleen: 5.5-fold at 96 hpi; liver: 9.1-fold at 3 hpi), and *IL-10* (head kidney: 10.5-fold at 3 hpi; spleen: 23.4-fold at 12 hpi; liver: 16.3-fold at 3 hpi) in *S. agalactiae*-treated group. Similar results were observed in pEGFP-C1+*S. agalactiae*-treated group and pEGFP-Gal8-L+*S. agalactiae*-treated group. Moreover, relatively weak increases of the examined cytokines were observed in the pEGFP-Gal8-L+*S. agalactiae*-treated group in comparison with pEGFP-C1+*S. agalactiae*-treated group. For instance, *IL-1*β was reduced at 3 and 6 hpi in head kidney, 12 hpi in spleen, and 3 and 6 hpi in liver; *TNF*α was down-regulated at 6, 12, and 24 hpi in head kidney, 72 hpi in spleen, and 3 hpi in liver; and *IL-10* was decreased at 48, 72, and 96 hpi in head kidney, 12, 72, and 96 hpi in spleen, and 3, 12, 48, and 72 hpi in liver.

### OnGal8-L Overexpression Improved the Survival Rates of Fish After *S. agalactiae* Infection

At day 7 after plasmid injection, the injected fish were challenged with *S. agalactiae* and the survival rates were evaluated. The results showed that the survival rate of the *S. agalactiae*-injected group was 20%, and the survival rate of the pEGFP-C1-injected group was 22.5%, while the survival rate of the pEGFP-Gal8-L-injected group was 52.5% (Figure 6D).

## Discussion

As a family of evolutionarily conserved carbohydrate-binding proteins, galectins can interact with glycoconjugates of cell surface and extracellular matrix, thereby participating extensively in innate and adaptive immune processes (35, 36). In this study, a new tandem-repeat galectin (OnGal8-L) from Nile tilapia (*Oreochromis niloticus*) was identified, and its roles in antibacterial immune response also were investigated. OnGal8-L encoded a 296-amino acid peptide that shared 46.42 and 45.57% identities with reported *Oreochromis niloticus* galectin-8 and *Homo sapiens* galectin-8, respectively. Further comparison of OnGal8-L with reported galectin-8 revealed that OnGal8-L protein has two characteristic CRDs with different linker length. Given that a difference in linker length was also found in two human galectin-8 isoforms produced by the alternatively spliced of LGALS8 (37), we assume that OnGal8-L and reported *Oreochromis niloticus* galectin-8 may be two alternatively spliced isoforms of galectin-8. This assumption needs to be clarified in future studies. Moreover, given CRD is vital to mediate cell–cell or cell–intercellular matrix interactions in developmental processes and immunity, the co-existence of two different CRDs increases the diversity of ligand recognition by tandem repeat galectin (38). The high sequence similarities in both the N- and C-CRD regions of OnGal8-L indicate that it may exerts similar functions as reported galectin-8.

The current data exhibits that the *OnGal8-L* transcripts are present in all examined tissues with highest level in spleen. Fish spleen is one of the major peripheral lymphoid organs in fish (39), this result indicates OnGal8-L may play roles in the lymphatic system. Additionally, the high transcriptional levels of *OnGal8-L* are observed in skin, brain, and intestines, which is similar to the findings of OnGal-2 and OnGal-9 in tilapia (17, 18). As organs contact the external environment, the skin and intestine of fish are considered to be the first line of defense against pathogens (40, 41), and the blood–brain barrier in brain can resist the penetration by *S. agalactiae* (42). The abundance of *OnGal8-L* in these tissues indicates that it may be associated with immune defense against pathogens. To confirm whether *OnGal8-L* is involved in immune response against bacterial infection, the temporal transcription expressions of *OnGal8-L* in different tissues (spleen, head kidney, and brain) are investigated by qRT-PCR. Our results show that *OnGal8-L* is up-regulated in a time-dependent manner and reached to peak at 12 h in spleen, head kidney, and brain after *S. agalactiae* infection, indicating that OnGal8-L plays a role in the anti-bacterial immune response of tilapia. Similar results are also observed in the studies of OnCL-K1 and OnGal-2 from Nile tilapia (17, 27). It has been proven that *S. agalactiae* can invade the brain microvascular endothelial cell (BMEC) of fish, leading to meningitis (43). The increased expression of *OnGal8-L* in brain implies its contribution in protecting self-tissue. Compared with the transcriptional level in brain, a stronger increase of *OnGal8-L* was seen in spleen and head kidney at 12 hpi. Spleen and head kidney are the two main immune organs in teleost (44), which are armored with various immune cell types, including B cells, macrophages, granulocytes, and T cells. These results suggest that OnGal8-L may be involved in the regulation of cell-mediated immune responses during bacterial infection. Moreover, monocyte/macrophages are vital leukocytes that modulate innate immune responses through antigen presentation and cytokine production (45, 46). In our present data, we also observed the up-regulation of *OnGal8-L* in monocyte/macrophages after *S. agalactiae* stimulation, which is in line with the result of mannose-binding lectin in monocyte/macrophages (47). The *in vivo* and *in vitro* data indicate an involvement of OnGal8-L in response to bacterial infection.

To explore the exact function of OnGal8-L, the recombinant protein (rOnGal8L) was prepared for *in vitro* study. rOnGal8L exhibits the hemagglutination activity toward fish erythrocyte and bacterial, which is consistent with the research of Madusanka et al. (12), and the binding of rOnGal8L to *S. agalactiae* is impaired by competitive binding of LTA. Also, rOnGal8L can bind to LTA, LPS, poly I:C, PGN, galactose, mannose, and maltose, suggesting its utility in recognizing a wide range of pathogens. Besides the agglutination and binding activities, antibacterial activities are observed in a few galectin family members as well (48). Interestingly, our data showed that rOnGal8L significantly suppressed the growth of *S. agalactiae* but not *A. hydrophila*, which could be attributed to the different cell wall compositions of *S. agalactiae* (Gram-positive) and *A. hydrophila* (Gram-negative). These data indicate that rOnGal8-L acts as a PRR or an antimicrobial molecule in the immune defense against bacteria.

Considering that galectins recognize molecular patterns on the surface of microbes and initiate the downstream immune signaling pathways for bacterial clearance (49, 50), we further examined the impacts of rOnGal8L on phagocytosis and cytokine production of monocyte/macrophages. As expected, rOnGal8L can remarkably enhance the phagocytosis of macrophage to both Gram-positive and Gram-negative bacteria, which is in line with the finding of galectin-2 in tilapia (17). Respiratory burst is crucial for degrading internalized particles and bacteria in phagocytes. It has been recorded that fish lectins, including C-type lectin (27), galectin (18), and rhamnose-binding lectin (51), can enhance the respiratory burst activity of monocyte/macrophages. Consistently, stronger respiratory burst of macrophage was detected after rOnGal8L treatment in this study, indicating a role of OnGal8-L as opsonin during pathogen invasion. Apart from phagocytosis, monocytes/macrophages can mediate immune response by producing a variety of cytokines such as interleukin 6 (*IL-6*), interleukin 10 (*IL-10*), interleukin 8 (*IL-8*), and macrophage migration inhibitory factor (*MIF*) (52, 53). Similar to the finding of Mu et al. (54), our present study found that *IL-6* and *IL-10* rose significantly and peaked at 12 h after rOnGal8L incubation (Figure 4Aa,b). *IL-6* is a pleiotropic pro-inflammatory cytokine that can be rapidly expressed and secreted in the immune regulation process (55), while *IL-10* inhibits the pro-inflammatory response and limits unnecessary tissue destruction caused by inflammation (56). The increase of these two cytokines in the early stages of immune response suggests that OnGal8-L can activate inflammatory response of monocyte/macrophages. In addition, relatively delayed response patterns of *IL-8* and *MIF* were observed in rOnGal8L-treated group (Figure 4Ac,d), which slowly increased and reached to the highest level at 24 h. The results were consistent with our previous finding of OnGal-3 (32). As a neutrophil chemokine, IL-8 can recruit different immune cells including macrophages, mast cells, neutrophils, and other granulocytes to the infection sites (57). MIF, a macrophage migration inhibitory factor, which is necessary for leukocytes to transport to infection site (58). The observation implies that OnGal8-L may not only stimulate the inflammatory factors expression, but also induce the chemokines production for recruiting leukocytes to the infection site during antibacterial defense.

In fish, non-specific immunity is required for maintaining dynamic balance, preventing microbial invasion, eliminating various pathogens, and activating adaptive immune responses (59). AKP functions as a multi-functional enzyme participates in antimicrobial activity (60). ACP is a typical lysosome enzyme involved in killing and digesting pathogens (61). LZM also has potent non-specific antimicrobial activity (62). Moreover, as a component of antioxidant defenses, enzymatic antioxidants can protect fish against oxidative damage (63, 64). CAT, SOD, and POD are the primary antioxidant enzymes that represent neutrophil antimicrobial activity (65, 66). SOD can convert oxygen radicals (O^2−^) into H_2_O_2_, which is subsequently disproportionated or decomposed by CAT and POD (67, 68). The present study found that OnGal8-L overexpression enhanced the activities of AKP, ACP, LZM, CAT, POD, and SOD in serum, which clearly suggest that OnGal8-L can improve non-specific immunity and antioxidant activity in tilapia. The subsequent challenge experiment showed that overexpression of OnGal8-L also decreased bacterial burden and tissue damage in head kidney, spleen, and liver after *S. agalactiae* infection. Additionally, OnGal8-L overexpression reduced the expression of *IL-1*β, *TNF-*α caused by bacterial infection. While a significant up-regulation of *IL-10* was seen in the late stage of bacterial infection, indicating OnGal8-L can regulate inflammation response *in vivo* during bacterial infection. After *S. agalactiae* infection, the pEGFP-Gal8-L injected group had a better survival rate than the other groups, which suggests that OnGal8-L may be promising for applying as a molecular adjuvant in the development of vaccine against bacterial infection.

In summary, a novel tandem-repeat fish galectin-8 homolog (OnGal8-L) and its involvement in immune response during bacterial infection were identified in Nile tilapia. The *OnGal8-L* was widely distributed in various tissues and was induced by bacterial infection *in vivo* or *in vitro*. OnGal8-L could protect tilapia against bacterial infection via stimulating immune response, inhibiting bacterial proliferation and impairing tissue damages. This study lays a foundation for further study of fish galectin-8 in modulating immunity and sheds light on its potential application as vaccine adjuvant.

## Data Availability Statement

The raw data supporting the conclusions of this article will be made available by the authors, without undue reservation, to any qualified researcher.

## Ethics Statement

The animal study was reviewed and approved by Guangdong Province Laboratory Animal Management Regulations.

## Author Contributions

JC and YH designed the experiments. JN performed experiments, analyzed data, and wrote the manuscript. XL and FW performed Nile tilapia farming and cell isolation. BW and JT contributed to the graphing. JJ and YL reviewed the manuscript. All authors contributed to the article and approved the submitted version.

## Conflict of Interest

The authors declare that the research was conducted in the absence of any commercial or financial relationships that could be construed as a potential conflict of interest.
